# Rebound in community antibiotic consumption after the observed decrease during the COVID-19 pandemic, EU/EEA, 2022

**DOI:** 10.2807/1560-7917.ES.2023.28.46.2300604

**Published:** 2023-11-16

**Authors:** Cèlia Ventura-Gabarró, Vivian H Leung, Vera Vlahović-Palčevski, Anna Machowska, Dominique L Monnet, Liselotte Diaz Högberg, ESAC-Net study group, ESAC-Net study group participants, Reinhild Strauss, Lucy Catteau, Ivan Stoikov, Marina Payerl-Pal, Isavella Kyriakidou, Michal Prokeš, Majda Attauabi, Janne Sepp, Emmi Sarvikivi, Karima Hider-Mlynarz, Birgitta Schweickert, Kassiani Mellou, Maria Matuz, Anna Margrét Halldórsdóttir, Emre Umut Gurpinar, Filomena Fortinguerra, Ieva Rutkovska, Rolanda Valinteliene, Stéphanie Saleh, Peter Zarb, Jan Baltink, Hege Salvesen Blix, Anna Olcza-Pieńkowska, Ana Silva, Gabriel Adrian Popescu, Tomáš Tesař, Maja Subelj, Antonio López-Navas, Ragda Obeid

**Affiliations:** 1 European Centre for Disease Prevention and Control, Solna, Sweden; 2 Clinical Hospital Center Rijeka and University of Rijeka Medical Faculty, Rijeka, Croatia; 3 The members of the ESAC-Net study group are listed in the Collaborators tab

**Keywords:** Antimicrobial consumption, antibiotic consumption, EU/EEA, COVID-19 pandemic

## Abstract

We observed a rebound in consumption of antibacterials for systemic use (ATC J01) in the community sector in the European Union/European Economic Area during 2021 and 2022, after an observed decrease between 2019 and 2020. The rates in 2022 returned to pre-COVID-19-pandemic levels and were exceeded in 13 countries. Although these patterns could partly be a result of changes in disease transmission during the study period, it could also reflect a lost opportunity to strengthen and reinforce prudent antibiotic use.

In 2020, coinciding with the first year of the COVID-19 pandemic, the European Union (EU)/European Economic Area (EEA) saw a significant decrease in antibiotic consumption in the community sector [1]. However, this decrease appears to be transient, with EU/EEA-level being back at pre-pandemic levels in 2022 [2].

To better understand these changes, and specifically the timing and magnitude of the rebound period in 2021 and 2022, we analysed community sector consumption of antibacterials for systemic use group (anatomical therapeutic chemical (ATC) group J01), quantified as defined daily doses (DDD) per 1,000 inhabitants per day (ATC/DDD index for 2023 [3]), and as reported to the European Surveillance of Antimicrobial Consumption Network (ESAC-Net) [4].

## Variations in total community antibiotic consumption between 2019 and 2022

The Council Recommendation adopted in June 2023 on stepping up EU actions against antimicrobial resistance in a One Health approach (2023/C 220/01) [5] set an EU target of a 20% reduction in total antibiotic consumption (community and hospital sectors combined) by 2030, using 2019 as the baseline year. As around 90% of the total consumption represents community consumption [2], considerable and consistent reductions in this sector are essential to reach the EU target. 

**Figure 1:**
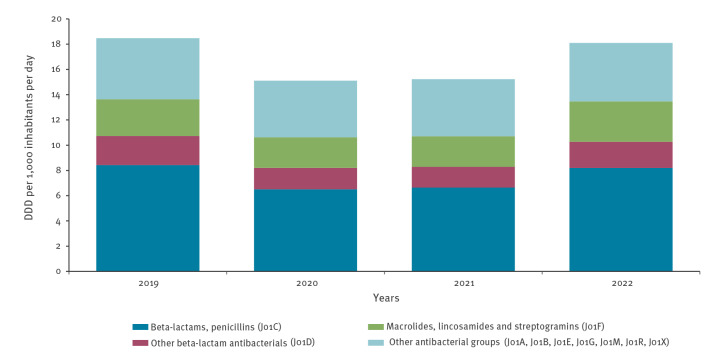
Community consumption of antibacterials for systemic use (ATC group J01) by ATC group, population-weighted mean of 26 EU/EEA countries, 2019–2022

ATC: anatomical therapeutic chemical classification; DDD: defined daily doses; ECDC: European Centre for Disease Prevention and Control; EEA: European Economic Area; EU: European Union; TESSy: The European Surveillance System.

**Figure 2:**
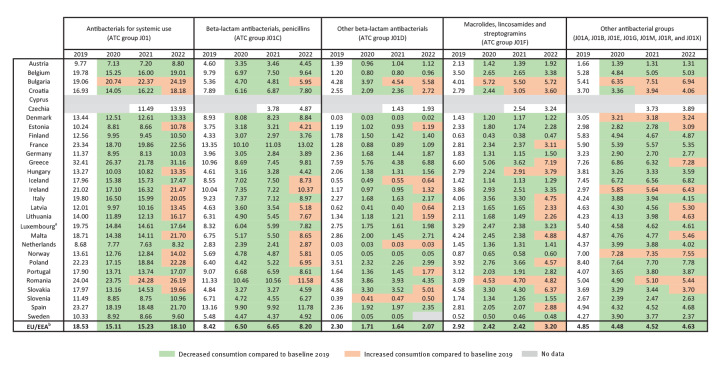
Community consumption of antibacterials for systemic use (ATC group J01) expressed as DDD per 1,000 inhabitants per day, 29 EU/EEA countries, 2019–2022

ATC: anatomical therapeutic chemical; DDD: defined daily doses; ECDC: European Centre for Disease Prevention and Control; EEA: European Economic Area; EU: European Union; TESSy: The European Surveillance System.

^a^ Luxembourg changed the data process between 2019 and 2020, which could impact comparability between the years.

^b^ The population-weighted mean is based on antimicrobial consumption data reported by 26 EU/EEA countries with complete data at ATC level 3 for the community sector for the all years 2019 to 2022 (Austria, Belgium, Bulgaria, Croatia, Denmark, Estonia, Finland, France, Germany, Greece, Hungary, Iceland, Ireland, Italy, Latvia, Lithuania, Luxembourg, Malta, the Netherlands, Norway, Poland, Portugal, Romania, Slovakia, Slovenia, and Spain). Sweden did not report data at ATC level 3 in 2022 and was excluded from the EU/EEA mean. The population-weighted mean was calculated by multiplying the DDD per 1,000 inhabitants per day for each country with the corresponding Eurostat population and dividing the product by the total population of all participating countries contributing data for the same year.

However, between 2019 and 2022, we observed a high variability in the EU/EEA population-weighted mean community antibiotic consumption (Figure 1), in contrast to a continuously slow decrease observed between 2015 and 2019 [2]. Although there was an unprecedented 18.5% decrease in community consumption in 2020 compared with the 2019 baseline [1], this decrease appeared to be transient. Between 2021 and 2022, the EU/EEA mean community consumption increased by 18.8% and showed no significant difference from the pre-pandemic level in 2019 (paired t-tests with individual country data points, p = 0.526). Moreover, in 13 of 27 countries, community antibiotic consumption was higher in 2022 than in 2019, with an average increase of 8.4% among these 13 countries (range: 0.6–26.9) (Figure 2).

The population-weighted mean is based on antimicrobial consumption data reported by 26 EU/EEA countries with complete data at ATC level 3 for the community sector for the all years 2019 to 2022 (Austria, Belgium, Bulgaria, Croatia, Denmark, Estonia, Finland, France, Germany, Greece, Hungary, Iceland, Ireland, Italy, Latvia, Lithuania, Luxembourg, Malta, the Netherlands, Norway, Poland, Portugal, Romania, Slovakia, Slovenia and Spain). Sweden did not report data at ATC level 3 in 2022 and was excluded from the EU/EEA mean. The population-weighted mean was calculated by multiplying the DDD per 1,000 inhabitants per day for each country, with the corresponding Eurostat population and dividing the product by the total population of all participating countries contributing data for the same year.

Data extracted from ECDC TESSy on 4 October 2023.

Values may differ from those in the ECDC Annual Epidemiological Reports on Antimicrobial Consumption due to differences in the included countries.

Data extracted from ECDC TESSy on 4 October 2023.

## Timing of the rebound to pre-pandemic consumption levels

**Figure 3:**
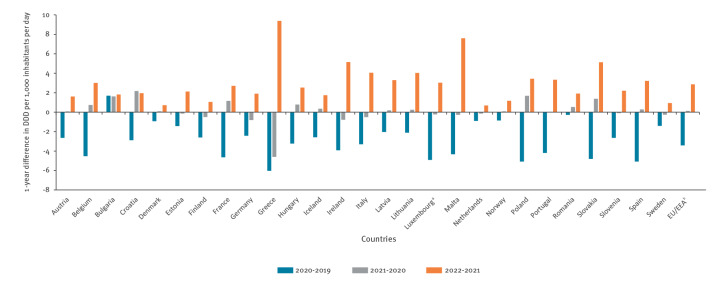
One-year differences in community consumption of antibacterials for systemic use (ATC group J01) in the community, 27 EU/EEA countries, 2019–2022

ATC: anatomical therapeutic chemical; DDD: defined daily doses; ECDC: European Centre for Disease Prevention and Control; EEA: European Economic Area; EU: European Union; TESSy: The European Surveillance System.

^a^ Luxembourg changed data process between 2019 and 2020, which could impact comparability between the years.

^b^ EU/EEA: The population-weighted mean is based on antimicrobial consumption data reported by 26 EU/EEA countries with complete data at ATC level 3 for the community sector for the all years 2019 to 2022 (Austria, Belgium, Bulgaria, Croatia, Denmark, Estonia, Finland, France, Germany, Greece, Hungary, Iceland, Ireland, Italy, Latvia, Lithuania, Luxembourg, Malta, the Netherlands, Norway, Poland, Portugal, Romania, Slovakia, Slovenia and Spain). Sweden did not report complete data at ATC level 3 in 2022 and was excluded from the EU/EEA mean. The population-weighted mean was calculated by multiplying the DDD per 1,000 inhabitants per day for each country with the corresponding Eurostat population and dividing the product by the total population of all participating countries contributing data for the same year.

In the EU/EEA overall, as in 15 individual countries (Austria, Denmark, Estonia, Iceland, Latvia, Lithuania, Luxembourg, Malta, the Netherlands, Norway, Portugal, Romania, Slovenia, Spain and Sweden), there was no or marginal (less than +/−3%) change in antibiotic consumption between 2020 and 2021, while a rebound, that is, going back to the pre-pandemic levels of 2019, was apparent between 2021 and 2022 (average increase: 20.5%, range: 5.7–53.9). The rebound occurred earlier between 2020 and 2021 for six countries (Belgium, Croatia, France, Hungary, Poland and Slovakia, average increase: 7.9%, range: 4.9–10.5). In contrast, five countries (Finland, Germany, Greece, Ireland and Italy) reported a continued decrease in antibiotic consumption between 2020 and 2021 (average decrease: −7.9%, range: −3.1 to −17.4) before a rebound in 2022 (Figure 3) (average increase: 26.9%, range: 11.1–43.1). Notably, consumption level in Greece continued to decline in 2021 nearly at the same rate as in 2020, followed by the largest rebound among all countries (annual changes from 2019 to 2022 of −18.6%, −17.4% and +43.1% respectively). Inversely, Bulgaria reported a continuous, regular increase during the period 2019 to 2022 (annual increases of 8.9%, 7.8% and 8.13% respectively) (Figures 2 and 3).

Data extracted from ECDC TESSy on 4 October 2023.

## Increased consumption of macrolides, lincosamides and streptogramins (ATC group J01F)

At the EU/EEA level, there were no significant differences in 2022 consumption compared with the pre-pandemic quantities in 2019 for any of the antibiotic groups except for ‘macrolides, lincosamides and streptogramins’ (ATC group J01F), for which there was a statistically significant higher consumption in 2022 than in 2019 (p = 0.029). For individual countries, a more heterogeneous pattern was observed, with 13 countries reporting a higher consumption of macrolides, lincosamides and streptogramins in 2022 than in 2019 (Figure 2). Of these 13 countries, 10 also showed an overall increase of antibacterials for systemic use (ATC group J01). However, macrolide consumption was not necessarily the sole driver of the increase in consumption for all these countries.

## Discussion

The unusual fluctuations seen in the EU/EEA antibiotic consumption between 2019 and 2022 highlight the need to better identify and understand the factors influencing antibiotic consumption in a pandemic context. The initial decrease in antibiotic consumption during the first phase of the COVID-19 pandemic has been attributed to non-pharmaceutical interventions generally reducing the spread of pathogens [1,6,7], as well as disrupted access to healthcare services influencing prescription practices [8]. Between 2020 and 2022, most countries gradually lifted non-pharmaceutical interventions [9]. Our data show that the decrease in community antibiotic consumption in the EU/EEA was also transient, as consumption in 2022 went back to pre-pandemic levels.

Although the resurgence of both viral and bacterial respiratory tract infections during the latter part of our study period [10-14] might partly explain this rebound in antibiotic consumption, the increase could also reflect a missed opportunity to strengthen and reinforce prudent antibiotic use. The Council Recommendation set an EU target of a 20% reduction in total antibiotic consumption (community and hospital sectors combined) by 2030, using 2019 as the baseline year (2023/C 220/01) [5]. Although such a reduction had almost been achieved in 2020 at a community level due to factors related to the COVID-19 pandemic [15], the rebound in 2022 moved antibiotic consumption rates back towards the 2019 baseline value. To reach the recommended reduction targets by 2030, the EU and most EU countries will need to intensify their efforts to reduce unnecessary antibiotic consumption. Antibiotic stewardship activities strengthening prudent community consumption plays a vital role in this, as community consumption accounts for around 90% of the total antibiotic consumption [2].

There is a lack of patient-level information including prescription indications and actual use of antibiotics in ESAC-Net data, which derive mainly from sales and reimbursement records. Therefore, it is not possible to determine to which extent the observed rebound effect was driven by disease patterns or changes in behaviours. Nonetheless, observed variations across countries, both in the reduction of antibiotic consumption in 2020 and the subsequent rebound, suggest variations in potential inappropriate antibiotic use across countries. The 13 countries in which the rebound effect led to higher antibiotic consumption in 2022 compared with 2019, as well as the countries with the most ambitious reduction targets set out in the EU Council recommendation, now need to intensify measures to reduce unnecessary antibiotic use.

Although the overall community antibiotic consumption increased from 2021 to 2022 in all studied EU/EEA countries, there was variation in the start of rebounds before 2022 and in the antibiotic subgroups involved in the rebounds. These differences suggest that the drivers of antibiotic consumption are country-specific, which might lead to variations in their trajectories of antibiotic consumption.

Variations in consumption between 2019 and 2022 were also observed at antibiotic subgroup level, which reflects the complexity of reducing antibiotic consumption. At the EU/EEA level, the surge in consumption of ‘macrolides, lincosamides and streptogramins’ (ATC group J01F) in 2022 was largely driven by macrolide consumption [2], and more particularly azithromycin (data not shown). Following claims in the media that azithromycin could be used to treat COVID-19, its consumption increased in some European countries, in particular during the first months of the COVID-19 pandemic [16-18]. It is also possible that macrolides were more frequently used during the shortages of penicillins reported during 2022 [19,20], which could potentially also explain the increased consumption of ‘other beta-lactam antibacterials’ (J01D, which includes cephalosporins) in some countries in 2022. Antibiotic stewardship, including updated and evidence-based treatment guidelines, remain essential to address these issues.

## Conclusions

The COVID-19-pandemic had a substantial impact on community antibiotic consumption in the EU/EEA between 2020 and 2022. Countries exhibited different patterns of antibiotic consumption, underlining the importance of understanding each country in its own context. Further examination into local prescribing and consumption behaviours for specific antibiotic groups can inform effective stewardship interventions and bring the EU/EEA closer to its antibiotic consumption targets for 2030.
