# Author’s correction for Euro Surveill. 2023;28(46)

**DOI:** 10.2807/1560-7917.ES.2023.28.48.231130c

**Published:** 2023-11-30

**Authors:** Eurosurveillance editorial team

**Affiliations:** 1 European Centre for Disease Prevention and Control (ECDC), Stockholm, Sweden

In the article entitled ‘Rebound in community antibiotic consumption after the observed decrease during the COVID-19 pandemic, EU/EEA, 2022’ by Ventura-Gabarró et al. published on 16 November 2023, the name of collaborating author Anna Olczak-Pieńkowska was misspelled as Olcza-Pieńkowska in the originally published version. This was corrected on 30 November 2023 at the request of the authors.

